# Retaining Diabetic Eye Exams in Medicare Advantage Star Ratings: A Policy Commentary on Prevention, Equity, and Quality Measurement

**DOI:** 10.7759/cureus.110303

**Published:** 2026-06-05

**Authors:** Hossein Mousazadeh, Dean VanNasdale, Rajeev S Ramchandran

**Affiliations:** 1 Department of Ophthalmology, Flaum Eye Institute, University of Rochester Medical Center, Rochester, USA; 2 Department of Environmental Medicine and Public Health, University of Rochester Medical Center, Rochester, USA; 3 College of Optometry, The Ohio State University, Columbus, USA

**Keywords:** cms star ratings, diabetic retinopathy, health equity, preventive eye care, quality measurement

## Abstract

In 2025, the Centers for Medicare and Medicaid Services (CMS) proposed removing the diabetic eye examination measure from the Medicare Advantage Star Ratings program, raising concern about the future visibility of preventive vision care within chronic disease management. Following public comment and stakeholder engagement, CMS retained the measure in the 2026 final rule. This editorial examines the policy implications of that decision for quality measurement, prevention, health equity, and value-based care. Diabetic retinopathy remains a major and growing cause of preventable vision loss in the United States, with important implications for Medicare beneficiaries, underserved populations, and health system costs. Although the diabetic eye examination measure presents challenges related to attribution, care coordination, and follow-up, these challenges reflect systemic barriers that require stronger implementation strategies rather than removal from accountability frameworks. Retaining the measure signals the continued importance of prevention in diabetes care and creates an opportunity to modernize quality measurement through integrated, equitable, and technology-enabled models that address screening disparities, referral completion, comprehensive diabetic eye care, and closed-loop follow-up.

## Editorial

Introduction

Vision loss is well established as a major public health challenge with a growing global burden and substantial societal impact [1], yet it remains unrecognized as a healthcare priority, and institutional responses are often fragmented and insufficient [2]. In the United States, vision loss carries profound implications for functional independence, quality of life, and health system costs, reflecting a broader global burden of largely avoidable visual impairment [3]. Among its most preventable causes, diabetic retinopathy continues to account for substantial morbidity; in 2021, an estimated 9.60 million people in the United States had diabetic retinopathy, including 1.84 million with vision-threatening diabetic retinopathy [4]. This burden is expected to grow, with projections estimating that diabetic retinopathy may affect 16.0 million Americans aged 40 years or older by 2050. Also, it is expected to grow as the prevalence of diabetes rises, with important implications for Medicare beneficiaries, health systems, and payers. In addition to its clinical burden, diabetic retinopathy carries substantial economic implications, as delayed detection and treatment can increase the need for more intensive downstream care, while US-based economic analyses support the value of diabetic retinopathy screening and treatment strategies.

In 2025, the Centers for Medicare and Medicaid Services (CMS) proposed removing the diabetic eye exam measure from the Medicare Advantage Star Ratings Program, a national quality rating system used to evaluate health plan performance across preventive care, chronic disease management, and patient experience. The diabetic eye exam measure assesses whether beneficiaries with diabetes receive recommended diabetic eye examinations within specified time intervals.

This measure has helped keep eye health visible within chronic disease management. However, the completion of diabetic eye examinations remains difficult due to practical barriers in service coordination and care delivery. Current national progress toward improving annual diabetic eye exam completion remains limited, underscoring that measurement alone is insufficient. Rather than weakening the rationale for the measure, this lack of progress highlights the need to pair accountability with stronger implementation strategies that address access, coordination, and follow-up. As a systemic disease affecting multiple organ systems, diabetes management is challenging due to the need for multidisciplinary coordination across primary care, endocrinology, ophthalmology, and other health services [5]. Eliminating a quality metric that promotes diabetic eye health could make progress more difficult at a time when diabetes prevalence is rising, disparities in eye care access persist, and health systems are increasingly judged on value and outcomes. Sustained attention to completing diabetic eye exams may serve a valuable preventive and equity-oriented function. This is particularly important given documented racial and ethnic disparities in vision-threatening diabetic retinopathy, with higher prevalence reported among Black and Hispanic individuals compared with White individuals.

Following extensive public comment, CMS reversed course and retained the measure in its 2026 final rule. This decision represents more than a technical adjustment; it illustrates how quality metrics function as policy instruments that signal priorities, shape investment, and determine whether prevention remains visible within health systems. This policy reversal underscores the responsiveness of federal health policy to stakeholder engagement and reflects the cumulative impact of broad-based advocacy across clinical and public health communities. It demonstrates that quality measurement is not static but can be shaped through iterative dialogue between policymakers and the health system. Because this editorial is policy-oriented, we distinguish between the clinical evidence supporting diabetic eye care and our policy interpretation that retaining the measure helps maintain accountability and visibility for preventive vision care.

Policy case study: from proposed removal to policy reversal

The proposed removal of the diabetic eye exam measure sparked widespread response from clinicians, researchers, advocacy organizations, and community-based screening programs. Stakeholders emphasized that the measure has long served as a critical lever for promoting early detection of diabetic retinopathy, supporting care coordination, and incentivizing investment in screening and referral infrastructure that facilitates completion of recommended diabetic eye care. Others acknowledged limitations in the metric, including challenges related to attribution, data capture, and evolving care delivery models. This editorial does not present new empirical evidence that the Star Ratings measure alone increases diabetic eye examination rates; rather, it interprets the measure as a policy and accountability mechanism that can help maintain attention to preventive vision care when paired with stronger implementation strategies.

In its final rule, CMS explicitly recognized the importance of these perspectives and ultimately chose to retain the measure. The agency affirmed that “routine retinal screening is a critical component of comprehensive diabetes care” and highlighted its role in preventing vision loss, reducing downstream costs, and preserving functional independence. CMS further emphasized that maintaining the measure aligns with the broader goals of the STAR Ratings program, including promoting preventive care, supporting whole-person health, and advancing equitable access-particularly for high-risk and underserved populations.

Why this decision matters: prevention, value, and health system signals

Quality measures do more than assess performance-they define priorities. Within the STAR Ratings framework, inclusion of a measure directs attention, resources, and innovation. The diabetic eye exam measure, which assesses whether beneficiaries with diabetes receive recommended diabetic eye examinations, has historically helped keep vision health visible within chronic disease management. However, we do not argue that the measure alone has been sufficient to substantially improve eye exam completion rates. Rather, its value lies in maintaining accountability for a clinically important preventive service and signaling the need for stronger implementation strategies, including care coordination, patient navigation, teleophthalmology, and closed-loop referral systems. Persistently suboptimal examination rates, therefore, should not be interpreted as a reason to remove the measure but as evidence that the measure should be modernized and paired with system-level interventions to address barriers in access, delivery, and follow-up.

Regular eye examinations for people with diabetes are an important component of diabetic retinopathy prevention and management. Preventable vision loss can diminish independence, employability, mental health, and overall quality of life for individuals and their families [6]. Evidence suggests that regular eye examinations are associated with better visual and functional outcomes, supporting the role of timely detection and follow-up in preventing avoidable vision loss. As ophthalmologists, optometrists, and population health researchers, we witness the consequences of delayed or missed eye care among people with diabetes whose vision loss may have been prevented or reduced through timely detection and coordinated follow-up to sight-saving treatment. Diabetic eye disease remains a major cause of irreversible vision loss among working-age adults in the United States, yet only about half of eligible individuals receive recommended diabetic eye care annually [7]. This gap reflects not a failure of clinical evidence, but structural barriers within the health system, including fragmented care pathways, limited access to eye care providers, transportation challenges, and gaps in follow-up.

By retaining the measure, CMS reinforces the importance of prevention as a cornerstone of value-based care. Preventing vision loss can reduce late-stage complications, long-term disability, and loss of independence-outcomes central to both patient well-being and health system sustainability. U.S.-based economic analyses have found diabetic retinopathy screening and treatment to be cost-effective, including evidence from telemedicine-based screening programs in underserved urban populations [8].

Health systems lesson: measuring complexity, not avoiding it

One of the central criticisms of the diabetic eye exam metric is that it holds primary care providers accountable for services they do not directly deliver. Unlike foot exams, which can be performed during a primary care visit, eye exams require referral to a separate specialty, coordination across providers, and navigation of multiple logistical barriers. These complexities contribute to lower performance rates and frustration among clinicians. However, this complexity is precisely why the measure is essential. It incentivized care coordination, referral pathways, and follow-up processes. Eliminating the measure would not resolve these challenges and may reduce the visibility of these barriers within quality improvement frameworks.

Quality improvement begins with measurement. When complex, system-dependent outcomes are removed from accountability frameworks, the incentives to address underlying barriers diminish. The diabetic eye exam measure highlights the need for shared accountability across health systems, payers, and providers. It highlights the importance of integrated care models that bridge primary and specialty care and support patients at every step of the care continuum.

Equity implications: protecting vulnerable populations

The preservation of the diabetic eye exam measure also carries important equity implications. Vision loss from diabetic retinopathy disproportionately affects individuals from historically underserved communities, including those with lower socioeconomic status, limited access to care, and a higher burden of chronic disease. These disparities are also evident in diabetic eye care utilization. Among Medicare Part B fee-for-service beneficiaries with diabetes, only 54.1% received an eye examination in 2017, with lower rates among Black and Hispanic patient beneficiaries than among White beneficiaries [4]. More recent multicenter U.S. data also show that Black patients with preexisting diabetic retinopathy, Hispanic and White patients, and patients living in rural communities were less likely to receive guideline-recommended diabetic eye care [7].

Barriers to eye care-such as transportation, cost, insurance coverage, geographic access, language access, health literacy, food or housing insecurity, and mental health burden-are not evenly distributed. Recent national data from adults with type 2 diabetes also show that food insecurity, housing insecurity, and mental health status are associated with a lower likelihood of visiting an eye care practitioner for diabetic retinopathy screening [9]. Rural-urban access differences further complicate this equity landscape. Patients living in rural communities often face longer travel distances, fewer local eye care providers, limited availability of specialty care, and greater transportation barriers, all of which can reduce the likelihood of timely retinal screening, comprehensive eye examinations, and follow-up. These geographic barriers may be especially consequential when abnormal screening results require referral to ophthalmology or retina specialists. Without explicit measurement and accountability, these disparities risk widening. CMS’s decision to retain the measure signals a commitment to ensuring that preventive services reach populations most at risk. Importantly, the agency also acknowledged that the measure can support innovation in screening approaches, particularly for underserved populations. This includes expanding access through community-based programs, mobile screening units, and integration of services within primary care settings.

From preservation to modernization: the path forward

While the decision to retain the diabetic eye exam measure is a critical step, it should not mark the endpoint of policy evolution. Rather, it presents an opportunity to modernize the metric to better reflect contemporary models of care. Advances in teleophthalmology and retinal imaging technologies have expanded the capacity for retinal screening beyond traditional specialty settings, with recent U.S.-based evidence demonstrating that teleretinal screening programs in community health centers can improve diabetic retinopathy screening completion [10]. However, retinal screening should be distinguished from a comprehensive diabetic eye examination. Screening programs can identify patients who may need further evaluation, but they do not replace comprehensive examination, diagnosis, treatment planning, or longitudinal management by eye care professionals. In the context of persistent workforce maldistribution and limited access to eye care providers in many regions, teleophthalmology may be an important strategy for expanding access to early detection and triage. Primary care clinics, community health centers, and mobile units can facilitate retinal screening in underserved areas, but screening alone is insufficient to prevent vision loss unless abnormal findings lead to timely referral completion, comprehensive eye examination, treatment when indicated, and closed-loop follow-up. Quality measures should evolve to recognize these approaches as a way to expand access to care while helping build the evidence needed to guide more effective and integrated care delivery.

In addition, future iterations of the measure should emphasize closed-loop care, ensuring that retinal screening leads to appropriate referral, comprehensive eye examination, treatment when indicated, and documented follow-up. Integrating data systems across providers and health plans will be essential for tracking outcomes and reducing loss to follow-up. Stratifying performance by equity-relevant factors can further align the measure with broader goals of health equity. Finally, accountability should be distributed across the care team, including health plans, primary care providers, and eye care specialists. Health plans, in particular, are well positioned to support patient navigation, reduce logistical barriers, and invest in infrastructure that facilitates access to care.

Conclusion

CMS’s decision to retain the diabetic eye exam measure offers a clear lesson for health systems: when a metric is challenging to achieve, the solution is not to remove it, but to increase effort, innovation, and creativity in how care is coordinated, delivered, and measured. Outcomes that depend on coordination, access, and longitudinal care are often the most difficult to capture-and the most consequential for patients.

Inclusion of the diabetic eye exam measure affirms that prevention remains central to value-based care. Its preservation signals that vision health should not be peripheral, but integral to chronic disease management and functional independence. As health systems increasingly orient toward outcomes, quality metrics must evolve to reflect modern care delivery while maintaining accountability for equitable access.

Removing complex measures such as the diabetic eye exam measure may make quality reporting easier, but it would also reduce accountability for preventive services that are difficult to coordinate and important for patients. Preventable blindness is not a marginal outcome. Preserving and strengthening this measure represents a commitment to prevention, equity, and the long-term sustainability of health systems.
